# Protein-Carbohydrate Interactions, and Beyond …

**DOI:** 10.3390/molecules200815202

**Published:** 2015-08-20

**Authors:** Kristof De Schutter, Els J. M. Van Damme

**Affiliations:** Lab Biochemistry and Glycobiology, Department of Molecular Biotechnology, Ghent University, Coupure links 653, Ghent B-9000, Belgium; E-Mail: Kristof.DeSchutter@UGent.be

Carbohydrates are ubiquitous and play an intriguing role inside the cell as well as on the cell surface. In addition to being part of glycan structures on glycoproteins, glycolipids or peptidoglycans, carbohydrates also occur as mono- and polysaccharides. Over the last decade, evidence has accumulated indicating that interactions between carbohydrates and particular proteins that recognize them play critical roles in many biological processes, including physiological as well as pathological functions in the cell. In many instances, the interaction between the protein and the carbohydrate is not a simple event but only a first step in a series of events and interactions, often leading to a complex signaling cascade. In this special issue we want to focus on the broader picture of the interactions in the cell that rely on protein-carbohydrate recognition. This book provides an anthology of the importance of protein-carbohydrate interactions in 17 original contributions. Through a compilation of research articles and reviews, diverse protein-carbohydrate interactions in the different kingdoms of life are highlighted.

Almost one third of all newly synthetized polypeptides in a cell are targeted to the endoplasmic reticulum (ER) and the majority of them will be modified by N-linked glycosylation. However, a large fraction of proteins that enter the secretory pathway fail to fold properly [1]. To maintain fidelity of the cellular functions, a system is required to ensure that only properly folded proteins are transported through the secretory pathway. To this end, the ER uses an elaborate surveillance system called the “ER quality control system”. This system facilitates folding and modification of polypeptides and retains misfolded or incompletely assembled oligomers, and, if misfolding persists degrades the proteins through ER-associated protein degradation (ERAD). A key element in this quality control is the presence of an N-linked glycan structure on the glycoprotein. The review by Slomińska-Wojewódzka and Sandvig [2] provides an overview on the recent data highlighting the involvement of N-glycans in protein folding and in the regulation of glycoprotein degradation by ERAD. The main focus of the contribution is on lectins and their functions in protein turnover. The review by Wang *et al.* [3] provides a concise overview of the ER quality control system and ER stress.

Glycosylation is one of the most important posttranslational modifications. The two main types of protein glycosylation are *N*- and *O*-glycosylation. In vertebrates the biosynthetic pathways for *O*-glycosylation are well known, but the regulation and molecular aspects of defects are only poorly understood. Knowledge from *O*-glycosylation dependent events in invertebrates might help to elucidate the factors regulating this process. The review by Staudacher [4] provides an overview on the mucin-type *O*-glycosylation in invertebrates.

Many interactions within the immune system involve specific carbohydrate structures and proteins that recognize and bind them. The processes in which these interactions take place are very diverse and can be part of the infection process or rather can be related to the defense response. Künzler [5] reviews the fungal innate defense strategy based on effector proteins that target glycan epitopes. The review by De Schutter and Van Damme [6] discusses the protein-carbohydrate interactions in the plant and animal immune system whereas Legentil *et al.* [7] focus on a specific interaction in the immune system between β-(1-3)-glucans and their receptors.

Since protein-carbohydrate interactions are of critical importance in numerous pathological processes, it is no surprise that these interactions are targeted in the development of therapeutics. Angiogenesis, the formation of new blood vessels, is not only essential in normal development but is also crucial in various pathological processes such as oncogenesis. The review by Chiodelli *et al.* [8] provides a comprehensive overview on angiogenesis and the role of heparin/heparin sulfate proteoglycans in this process and also discusses the potential therapeutic use. Several lectins bind specifically to glycan structures present on cancer cells, and therefore have an immense potential in cancer research. The research article by Peppa *et al.* [9] describes the characterization of two variants of a lectin from *Sclerotium rolfsii* that can bind glycan structures present on all cancer types and are able to suppress tumor growth.

Glycan structures on the surface of pathogenic microorganisms or viruses can be used as targets for pharmaceuticals. However, long-term exposure to carbohydrate-binding agents (CBAs) results in mutant forms lacking the targeted glycan structure. Férir *et al.* [10] present an overview of HIV-1 and its resistance to CBAs.

The heamagglutinin (HA) glycan binding selectivity of influenza viruses determines the host range and egg adaptation in vaccine production. The article by Carbone *et al.* [11] describes the characterization of the HA binding selectivity of an egg-adapted influenza strain. By integrating the experimental data from glycan binding assays with structure-recognition models they examined the impact of mutations in HA on vaccine design.

Protein-carbohydrate interactions are interesting targets, not only for the development of therapeutics, but also for the production and processing of food products. One example is the use of chitosan, a linear polysaccharide, in the processing of milk and dairy products. The addition of chitosan causes milk proteins to coacervate. Chen *et al.* [12] performed a proteomic analysis of chitosan induced aggregation of milk proteins.

Lectins present in food can interact with the epithelial surface of the intestines and can induce physiological effects in humans and other animals. Although some lectins have a beneficial effect, several lectins -mainly legume lectins- have a detrimental effect. Nemoto *et al.* [13] report the effect of Chum Salmon Egg Lectin (CSL3) using Caco-2 cell monolayers as a model for the small intestine. CSL3 treatment resulted in a rearrangement of the cytoskeleton, thereby opening tight junctions in the Caco-2 monolayer.

During fertilization, protein-carbohydrate interactions play a key role in multiple important processes. The review by Miwa [14] focuses on the role of carbohydrate-mediated interactions between the sperm and the female reproductive tract, and discusses the fertilization-suppressive action of dicalcin.

Although essential for numerous processes in the cell, ranging from fertilization to development and good functioning of the immune system, all these individual protein-carbohydrate interactions are rather weak. To compensate for their low affinity, most protein-carbohydrate interactions are multivalent which complicates the study of these interactions *in vivo*. Johnson *et al.* [15] present an overview on the current state of computer simulations and biophysical models to study protein-carbohydrate interactions. Alternatively, novel technologies are developed or existing ones are being improved to overcome the difficulties in studying protein-carbohydrate interactions. In the paper by Wolfenden *et al.* [16] a novel assay is described for the evaluation of lectin binding to multivalent carbohydrates. The existing ELISA assays for the study of protein-sugar binding were not applicable for the study of galectins. Modification of the existing technology enabled the elaboration of a novel assay to study carbohydrate interactions with galectin-1 and -3.

A limiting factor in the study of glycan structures in the cell is the lack of lectins that can recognize specific glycan structures for the identification/annotation of these glycans. The review by Hu *et al.* [17] provides an overview on the current state of the art for engineering existing lectins into proteins with new carbohydrate-binding activities.

An important tool to study the biological function(s) of human proteins is the use of animal models, based on the assumption that the biological functions of the mouse protein will mimic those of the human counterpart. However, the question remains if this is really the case. Rambaruth *et al.* [18] characterized mouse mincle and investigated whether it can be used as a reliable model for human mincle.

This special issue provides the readers with a selection of articles and reviews highlighting the diversity and importance of protein-carbohydrate interactions, and hopefully will inspire the readers not only to look at the specific protein-carbohydrate interaction but also to what is beyond…
